# Extracorporeal photopheresis for cancer immunotherapy-related gastrointestinal toxicity: emerging clinical evidence

**DOI:** 10.1136/jitc-2026-016774

**Published:** 2026-10-21

**Authors:** Saikat Mandal, Manideepa Maji, Arkadeep Dhali

**Affiliations:** 1 Nottingham Digestive Diseases Centre, Translational Medical Sciences, University of Nottingham, Nottingham, UK; 2 NIHR Nottingham Biomedical Research Centre, Nottingham University Hospitals NHS Trust, Nottingham, UK; 3 Hull York Medical School, University of Hull, Hull, UK; 4 Haematology, Hull University Teaching Hospitals NHS Trust, Hull, UK; 5 Imperial College London, London, UK; 6 School of Medicine, Dentistry and Biomedical Sciences, Queen’s University Belfast, Belfast, UK; 7 Academic Unit of Gastroenterology, Sheffield Teaching Hospitals NHS Foundation Trust, Sheffield, UK

**Keywords:** Immune Checkpoint Inhibitors, Colitis, Immunotherapy, T-Lymphocytes, Gastrointestinal toxicities, Extracorporeal photopheresis

## Abstract

Immune-mediated gastrointestinal toxicities of cancer immunotherapy can be severe, corticosteroid-refractory, and treatment-limiting. Extracorporeal photopheresis (ECP) may provide immunomodulation with less systemic immunosuppression. To evaluate the role of ECP in immune-mediated gastrointestinal toxicity, we searched MEDLINE, Embase and Scopus from inception through July 2026 for human studies in which ECP was used to treat gastrointestinal toxicity associated with immune-checkpoint inhibitors or immune-effector-cell therapies. Study characteristics, treatment regimens, clinical response, corticosteroid use, mucosal healing, recurrence, and safety were narratively synthesized. Among 1423 records, seven publications were identified: three case reports, two uncontrolled cohorts, one prospective non-randomized comparative study, and one CAR T-cell enterocolitis series containing a single ECP-treated patient. Across reports of immune-checkpoint-inhibitor-associated colitis, ECP was followed by clinical improvement, corticosteroid exposure reduction, and, where assessed, endoscopic or histological improvement. In the PRIA ECP study, complete resolution occurred in 11/12 ECP-treated colitis events and 13/16 colitis events treated with systemic immunosuppression (91% vs 81%; p=0.61). Across all organ toxicities, ECP was associated with lower cumulative corticosteroid exposure and fewer worsening events. No serious ECP-attributable adverse events were reported. ECP is a promising rescue or corticosteroid-sparing option for selected refractory gastrointestinal toxicities; however, efficacy remains unproven because evidence is limited, heterogeneous, and predominantly uncontrolled. Randomized controlled trials evaluating standardized clinical and endoscopic outcomes, corticosteroid-free remission, safety, and treatment burden are required.

 Immune checkpoint inhibitors (ICIs) and immune effector cell therapies have transformed the treatment of solid and hematological malignancies but can provoke inflammatory toxicities resembling autoimmune disease. Gastrointestinal toxicities of ICIs include immune-mediated diarrhea and colitis, enteritis, gastritis, and duodenitis. Immune effector cell-associated enterocolitis is a distinct, rare gastrointestinal toxicity reported after chimeric antigen receptor (CAR) T-cell therapy, predominantly with B-cell maturation antigen (BCMA)-directed products.^1^ The incidence of all-grade ICI-related colitis is approximately 1.3% with anti-PD-1/PD-L1 therapy, 9.1% with ipilimumab, and 13.6% with combined ipilimumab and nivolumab checkpoint blockade.^2^ The corresponding incidences of grade 3–4 colitis are approximately 0.9%, 6.8% and 9.4%, respectively.^2^ Severe disease may cause bleeding, dehydration, malnutrition, bowel perforation, and interruption of otherwise effective anticancer therapy. Current guidelines recommend withholding ICI therapy and initiating corticosteroids for clinically significant toxicity, with escalation to infliximab or vedolizumab when colitis is refractory.^3^ Some patients nevertheless have persistent inflammation or relapse during corticosteroid tapering. Prolonged systemic immunosuppression also causes metabolic and infectious complications, while its effect on antitumor immunity remains a clinical concern. A treatment that controls organ-specific inflammation without broad immune suppression is therefore attractive.

Extracorporeal photopheresis (ECP) involves leukapheresis, ex vivo exposure of mononuclear cells to 8-methoxypsoralen and ultraviolet-A light, and reinfusion of the photoactivated cells. These cells subsequently undergo apoptosis and are taken up by antigen-presenting cells. This process is thought to induce immunomodulatory changes involving dendritic cells, macrophages, and regulatory T-cell responses, thereby attenuating pathogenic effector immunity.^4 5^ Translational work in ICI-related colitis additionally implicates local adiponectin production and reduced activation of intestinal tissue-resident T cells, without evident loss of systemic antitumor activity.^5^ We, therefore, systematically reviewed the clinical evidence for ECP in cancer-immunotherapy-associated gastrointestinal toxicity.

We searched Ovid MEDLINE, Ovid Embase and Scopus from inception to July 2026. Controlled vocabulary and free-text terms covered ECP; ICIs, CAR T-cells, and other cancer immunotherapies; and gastrointestinal phenotypes, including diarrhea, colitis, enteritis, enterocolitis, esophagitis, gastritis, duodenitis, mucosal ulceration, and immune effector cell-associated enterocolitis. Original human studies were eligible when ECP was administered with the intention of treating an immune-mediated gastrointestinal toxicity. Clinical trials, prospective or retrospective cohorts, case series, and case reports were included. A clinical diagnosis without endoscopic confirmation remained eligible because ICI studies frequently report the combined entity ‘immune-mediated diarrhea and colitis’; diagnostic support was classified as clinical, endoscopic, or histological.

Reviews, guidelines, preclinical studies, trial registrations without results, reports in which ECP was not administered, and studies without separable gastrointestinal data were excluded. Multiple publications from the same cohort were linked, and the most complete report was retained for this review. The searches retrieved 1423 records (Scopus: 1345; MEDLINE: 8; Embase: 70). After removing 78 duplicates, 1345 unique records were screened, and 7 eligible publications were retained. Study design, phenotype, diagnostic confirmation, ECP schedule, concomitant treatment, clinical response, corticosteroid use, recurrence, safety, and oncological outcomes were extracted. The principal outcome was resolution or improvement of gastrointestinal toxicity; secondary outcomes included time to response, corticosteroid reduction, mucosal healing, recurrence, treatment-related adverse events, and disease control. Outcomes were interpreted at the study level, and colitis events were distinguished from those in individual patients when multiorgan toxicity was reported. Heterogeneity and possible cohort overlap precluded meta-analysis; results were synthesized narratively.

The seven publications comprised three single-patient reports, two uncontrolled cohort studies, one prospective non-randomized comparative study, and one multicenter CAR T-cell enterocolitis series in which one patient received ECP (table 1). The three ICI-colitis cases provided concordant but uncontrolled observations. Apostolova *et al* reported complete clinical, colonoscopic and histological remission following ECP in a patient with ICI-induced colitis refractory to glucocorticoids, infliximab and ciclosporin.^6^ In the Ruf *et al* case, colitis resolved approximately 10 weeks after ECP was introduced, but concurrent vedolizumab prevented attribution of benefit to ECP alone.^7^ Pásek *et al* described grade 4 pancolitis unresponsive to corticosteroids and two doses of infliximab; daily stool frequency fell from approximately 24 to 8 after the first ECP cycle and to 4 after the second, with resolution 2 weeks later.^8^

**Table 1:** Clinical evidence for extracorporeal photopheresis (ECP) in cancer-immunotherapy-associated gastrointestinal toxicity

Study	Design and population	ECP intervention	Comparator	Outcome with ECP	Comparator outcome
Apostolova *et al*^6^	Case report; steroid-refractory ICI colitis (n=1)	Two consecutive treatment days every 2–4 weeks for 8 months	None; previous glucocorticoids, infliximab and ciclosporin	Complete clinical, colonoscopic and histological remission; immunosuppression tapered without relapse	No durable response to previous treatment
Ruf *et al*^7^	Case report; steroid-dependent/refractory ICI colitis (n=1)	Weekly, then every 2 weeks; corticosteroids and vedolizumab continued	None	Colitis resolved after approximately 10 weeks; ECP-specific effect uncertain	Previous corticosteroid/infliximab responses were temporary
Pásek *et al*^8^	Case report; grade 4 steroid- and infliximab-refractory ICI pancolitis (n=1)	Two cycles, each on 2 consecutive days, 2 weeks apart	None; corticosteroids continued	Stools decreased from 24 to 8/day after cycle 1 and to 4/day after cycle 2; subsequent resolution	No adequate response to corticosteroids plus 2 infliximab doses
Braun *et al*^5^	Prospective multicenter phase 1b/2 single-arm study; ICI colitis (n=8)	Two consecutive days weekly for 4 weeks, then every 2 weeks; median 16 procedures	None	Complete clinical remission in 8/8 at week 12; endoscopic and histological improvement; steroids reduced	Not applicable
Thielmann *et al*^9^	Retrospective series; severe ICI colitis (n=4)	4 to 5 cycles, each on 2 consecutive days, weekly or fortnightly	None; concomitant treatment continued	All improved from CTCAE grades 3–4 to ≤1; steroid dose <5% of baseline by week 9	Previous corticosteroid/biological treatment failed or was not durable
Wein *et al*^10^	Prospective non-randomized PRIA study; colitis events: ECP n=12, systemic immunosuppression n=16	Six fortnightly cycles over 12 weeks; previous immunosuppression could continue	Investigator-selected systemic immunosuppression, predominantly infliximab	Complete resolution in 11/12 (91%) at last follow-up	Complete resolution in 13/16 (81%); p=0.61
Susanibar-Adaniya *et al*^1^	Retrospective multicenter CAR T-cell enterocolitis series (n=19); ECP-treated n=1	Ruxolitinib followed by ECP; regimen not reported	None; heterogeneous treatments	Patient improved; ECP-specific effect could not be separated from ruxolitinib	Not estimable

CAR T-Cell, Chimeric antigen receptor T-cell; CTCAE, Common Terminology Criteria for Adverse Events; ECP, Extracorporeal photopheresis; ICI, immune checkpoint inhibitor.

Braun *et al* enrolled 14 patients with several ICI toxicities in a prospective phase 1b/2 study, including eight patients with colitis. All eight patients achieved complete clinical remission at week 12; corticosteroids were reduced in all evaluable patients, and paired endoscopic and histological assessments showed reduced inflammation.^5^ One colitis relapse after week 12 responded to further ECP. The accompanying 11-patient mixed-toxicity real-world cohort produced nine complete and one partial response, although gastrointestinal outcomes were not fully separable. In the Thielmann retrospective series, all four patients with severe ICI colitis improved from Common Terminology Criteria for Adverse Events grades 3–4 to grade 1 or lower; across the six-patient cohort, the median corticosteroid dose fell to less than 5% of baseline by week 9.^9^ The final PRIA analysis provided the only comparative evidence. The analysis included 12 colitis events managed with ECP and 16 managed with systemic immunosuppression. At last follow-up, complete resolution had occurred in 11 of 12 events in the ECP group (91%) and 13 of 16 events in the systemic immunosuppression group (81%; p=0.61).^10^ Across all organ toxicities, response at week 12 was 94% with ECP and 81% with investigator-selected immunosuppression (p=0.85). ECP was associated with lower cumulative prednisolone-equivalent exposure (395 vs 1260 mg; p=0.03) and fewer worsening events (6 vs 15; p=0.003), although allocation was non-randomized and the groups differed in toxicity complexity and previous treatment.

Evidence after CAR T-cell therapy was limited to one ECP-treated patient among 19 with ciltacabtagene-autoleucel-associated enterocolitis. Symptoms improved after sequential ruxolitinib and photopheresis, preventing isolation of the ECP-specific effect.^1^ No included study reported a serious adverse event attributed to ECP.^5 9 10^ However, adverse-event reporting was inconsistent, and the small cohorts could not exclude uncommon complications.

Across case reports and small cohorts, ECP was followed by improvement in severe, heavily pretreated ICI colitis, corticosteroid reduction and, in Braun *et al*, objective endoscopic and histological improvement.^5–9^ In PRIA ECP study, colitis resolution rates did not differ significantly between ECP and systemic immunosuppression, whereas cumulative corticosteroid exposure and worsening events across all organ toxicities favored ECP.^10^ Taken together, these findings support evaluation of ECP as a rescue or steroid-sparing strategy rather than routine first-line treatment. However, overall confidence in the evidence remains very low because the available studies are small, heterogeneous and predominantly uncontrolled. Three publications were case reports,^6–8^ and the uncontrolled cohorts were small.^5 9^ ECP was generally introduced after multiple previous therapies and was sometimes continued with corticosteroids, infliximab, vedolizumab or ruxolitinib, making causal attribution difficult.^1 6–9^ Treatment schedules, response definitions and follow-up varied substantially. Although PRIA study was prospective, it was non-randomized, was not designed specifically for gastrointestinal toxicity and reported several key outcomes across pooled organ toxicities.^10^ Evidence for CAR T-cell-associated enterocolitis is particularly insufficient because only one patient received ECP after ruxolitinib.^1^

The 2026 NCCN guidance stratifies management according to toxicity severity and recommends withholding ICI therapy and initiating corticosteroids for clinically significant colitis, with escalation to infliximab or vedolizumab for corticosteroid-refractory disease. In selected patients whose colitis remains refractory to these agents, tofacitinib, ustekinumab and fecal microbiota transplantation have been reported as later-line options, although supporting evidence remains limited, and specialist multidisciplinary oversight is required.^3^ On the other hand, ECP additionally requires specialist apheresis facilities, reliable venous access, and repeated treatment visits;^4^ its feasibility and time to response may therefore limit use in fulminant disease. Future trials should use prespecified clinical and corticosteroid-free remission, time to response, recurrence and hospitalization, supported by endoscopic and histological outcomes when appropriate. Concomitant immunosuppression, infection, quality of life, treatment burden, ICI rechallenge and tumor control should be reported separately. Evidence from randomized controlled trials is required before ECP can be positioned relative to established therapies.

The PHOENIX-ECP trial (NCT07619898), which is currently not yet recruiting, is a randomized, controlled, open-label, multicenter phase 2 study comparing ECP with the best available therapy in patients with unresectable or metastatic melanoma who develop grade ≥2 ICI-related colitis or hepatitis with an inadequate response to corticosteroids.^11^ The trial will enroll patients with an Eastern Cooperative Oncology Group (ECOG) performance status of 0–2 and requires endoscopic evidence of inflammation in those with colitis. Its primary outcome is steroid-free response at week 12; secondary outcomes include time to and duration of response, complete toxicity resolution, cumulative and peak corticosteroid exposure, corticosteroid discontinuation, treatment-emergent adverse events, and melanoma outcomes, including progression-free and overall survival.

## Conclusions

Current evidence suggests that ECP may offer a promising and generally well-tolerated corticosteroid-sparing strategy for selected patients with corticosteroid-refractory or corticosteroid-dependent gastrointestinal toxicities. However, its efficacy remains uncertain and requires confirmation in adequately powered randomized controlled trials.
